# Evaluating the benefits of TNF-alfa inhibitor biosimilar competition on off-patent and on-patent drug markets: A Southern European analysis

**DOI:** 10.3389/fphar.2022.1031910

**Published:** 2022-12-16

**Authors:** Teresa Barcina Lacosta, Arnold G. Vulto, Isabelle Huys, Steven Simoens

**Affiliations:** ^1^ Department of Pharmaceutical and Pharmacological Sciences, KU Leuven, Leuven, Belgium; ^2^ Hospital Pharmacy, Erasmus University Medical Center, Rotterdam, Netherlands

**Keywords:** biosimilars, biological medicinal products, off-patent biologicals, Southern-Europe, TNF-alfa inhibitors, price competition, prescribing behavior

## Abstract

**Background:** The level of competition achieved following biosimilars market availability varies by country, care setting and molecule. Hence, biosimilars contribution to attaining price reductions and extended access to treatments can also vary.

**Objective:** The aim of this study is to capture market dynamics for tumor necrosis factor (TNF)-alpha inhibitors and competing molecules in Southern European markets (2011–2020), and to evaluate the benefits of the competition generated by the availability of biosimilars.

**Methods:** This study is based on a literature review examining market characteristics for TNF-alfa inhibitors and competing immunomodulator molecules, and on the quantitative analysis of market data for these molecules in Italy, Portugal and Spain.

**Results:** Following biosimilars availability in Italian, Portuguese and Spanish markets, there has been an expansion in the overall access to TNF-alfa inhibitor pharmaceuticals. Further, savings have been generated within the TNF-alfa inhibitors class even after the increased use of these molecules. However, the potential of infliximab, etanercept and adalimumab biosimilars to generate price competition outside of their own drug class appeared limited in the studied markets. Considering this limitation and that shifts towards on-patent and higher-cost therapies have occurred after TNF-alfa inhibitor biosimilars availability, the importance of investing in biosimilars development for still on-patent immunology biologics is emphasized.

**Conclusion:** This study highlights the need for policies that do not only seek higher utilization of biosimilars, but that also support a sustainable market for these products. This is expected to foster the future development of biosimilar medicines.

## 1 Introduction

The market entry of biological medicines has revolutionized care for patients diagnosed with immune-mediated inflammatory conditions. The timely access to targeted biologic disease-modifying therapies (e.g., tumor necrosis factor (TNF)-alfa inhibitors) has allowed to improve patients’ outcomes, by reducing disease activity and facilitating disease remission (Li et al., 2017; Calleja-Hernández et al., 2020). However, the biologics development and manufacturing process comes at a higher cost compared to chemically synthesized therapies. As the demand for high-cost biologic medicines increases and the growth of the biologics market accelerates, healthcare systems struggle to control pharmaceutical expenditure (OECD, 2020). The market access of biosimilar medicines, after the expiration of patents and other exclusivity rights for originator biologics, represents a cost-reduction opportunity for healthcare systems. In the context of the current COVID-19 pandemic, and considering the overall rise in public spending, it has become critical to seize this opportunity (IQVIA, 2020b; 2021a).

Besides complying with the same quality, efficacy, and safety standards as originator biologics (EMA, 2019), biosimilars often generate competition dynamics when entering the market, generally leading to lowered prices and savings. Global list price savings from biosimilars are expected to reach $285 billion, cumulatively in the period 2020 to 2025. This figure is likely to increase in the coming years, as biosimilar market shares expand and the economic impact of exclusivity losses for biologics rises (IQVIA, 2021a). However, the level of competition generated by biosimilars varies according to the country’s biologics market, the care setting, and the molecule (Rémuzat et al., 2017; Moorkens et al., 2019a; Moorkens et al., 2019b; Moorkens et al., 2020; Moorkens et al., 2021). In this context, biosimilars contribution to attaining price reductions and extended access to treatments also varies (IQVIA, 2020b). Moreover, despite savings achieved across Europe following biosimilars competition, the access to specific molecules remains low in various jurisdictions. For instance, Inotai et al. identified a multitude of barriers affecting patient’s access to biologics in a set of Central and Eastern European Countries (Inotai et al., 2020). These are, among others, regulatory, administrative and resource barriers (e.g., budget limits for the prescription of biologics, and limits in the number of patients eligible for reimbursement).

In 2018, oncology and immunomodulating pharmaceuticals represented the highest expenditure category (48% of total expenditure) within the group of products purchased *via* centralized procedures in Portugal. Since then, National Health Service’s expenditure (SNS: Serviço Nacional de Saúde) associated to the purchase of on-patent biologic immunomodulators such as secukinumab, ixekizumab and vedolizumab has been on the rise (SPMS, 2018). Similarly in Spain, the purchase of oncology and immunomodulating agents represented 46% of the total 2019 hospital pharmaceuticals spending (Ministerio de Sanidad, 2019a). Along the same line, 2020 data provided by AIFA’s Medicines Utilization Monitoring Centre (OsMed) on pharmaceuticals use in Italy showed that antineoplastic and immunomodulating medicines had the highest consumption and expenditure among the products purchased directly by public structures. Within the group of immunosuppressant and immunomodulating agents, patent-covered products accounted for 94.8% of the expenditure (AIFA, 2020a; c). Based on these data, the health administrations in Portugal, Spain and Italy have implemented measures to leverage TNF-alfa inhibitor biosimilars competition and to foster the prescription of ‘best-value’ biologics (i.e., lower-priced originator biologics or biosimilars) in the immunology area (MFE, 2021).

For an exhaustive understanding of the real impact of the market availability and use of biosimilars in Europe, one should consider country-, care- and product-specific scenarios. This study aims to capture market dynamics following TNF-alfa inhibitor biosimilars availability in a sample of countries representative of Southern Europe (i.e., Italy, Portugal, Spain). Here, biologics are primarily prescribed/dispensed at the hospital level and incur high costs for National Health Services (MFE, 2021). An added value of this study with respect to published literature is that the impact of TNF-alfa inhibitor biosimilars availability is not only studied within the TNF-alfa inhibitors class, but across competing therapeutic classes, including off-patent and on-patent molecules. Further we analysed indication-specific data in rheumatology, in order to capture drug utilization shifts within a specific therapeutic area.

## 2 Materials and methods

The study methodology was designed according to published research analyzing markets for TNF-alfa inhibitors prior to and following biosimilars availability (Moorkens et al., 2019a; Moorkens et al., 2019b; Moorkens et al., 2020; Moorkens et al., 2021). We first conducted a literature review to describe aspects specific to the immunomodulators and TNF-alfa inhibitors market in Italy, Portugal and Spain. These three countries are representative of Southern Europe, as they make up three-quarters of the population in this area (Eurostat, 2021). The scientific databases PubMed/Medline, Embase and Google Scholar were searched up to April 2022, based on combined searches including the terms: “biosimilars”, “biologics”, “originator biologics”, “reference biologics”, “off-patent biologics”, “TNF-alfa inhibitors”, “immunomodulators”, “infliximab”, “etanercept”, “adalimumab”, “certolizumab pegol”, “golimumab”, “market data”, “market (dynamics)”, “spending”, “expenditure”, “costs”, “savings”, “drug utilization/volume/consumption”, “consumption volume”, “sales volume”; and “Italy”, “Portugal” and “Spain”. Literature in English, Italian, Portuguese and Spanish was included in the analysis.

Apart from screening academic databases, we searched gray literature repositories within websites of Spanish, Italy and Portuguese health institutions (e.g., Italian Medicines Agency (AIFA), AIFA’s Medicines Utilization Monitoring Centre (OsMed), Portuguese National Authority of Medicines and Health Products (Infarmed), Portuguese Central Administration of the Health System (ACSS), Spanish Medicines Information Centre (CIMA), Spanish Medicines Agency (AEMPS), etc.). In the light of findings from the literature review, we then conducted a quantitative analysis of market data for TNF-alfa inhibitors and competing immunomodulator molecules in Italy, Portugal and Spain.

### 2.1 Market data analysis

Our analysis is based on consumption volume and National Health Services’ acquisition costs evolutions for TNF-alpha inhibitors, in relation to competing biologic (e.g., Interleukin (IL) -inhibitors) and targeted synthetic disease-modifying molecules (e.g., Janus kinase (JAK) -inhibitors). The inclusion of molecules in the analysis (n = 16; adalimumab, abatacept, anakinra, apremilast, baricitinib, certolizumab pegol, etanercept, golimumab, infliximab, ixekizumab, rituximab, secukinumab, tocilizumab, tofacitinib, ustekinumab, vedolizumab; See Supplementary Table S2) was based on a review of country-specific guidelines published by rheumatology, gastroenterology, and dermatology associations for immune-mediated inflammatory diseases (Buch et al., 2011; SER, 2014; Torres et al., 2016; Duarte et al., 2017; Gisondi et al., 2017; Lamb et al., 2019; Torres T. et al., 2020; AEG, 2021; FINISTERRA, 2021; NICE, 2021). For validation purposes, the information from these guidelines was compared with European guidelines (Nast et al., 2017; Torres J. et al., 2020; Smolen et al., 2020; Sriranganathan et al., 2021).

It is to be noted that the inclusion of molecules in the analysis has been affected by limitations in data gathering/reporting at the level of the National Health Agencies for the countries of study. Databases searched for Italy, Portugal and Spain reported consumption volume data in either defined daily doses (DDDs) or number of units (e.g., tablets, pens, injectable solutions). Acquisition cost data for the three countries of study are based on National Health Service’s expenditure (in euros) and do not account for confidential discounts or rebates following tender negotiations. Volume and cost data were provided by the Portuguese National Authority of Medicines and Health Products (Infarmed) and the Spanish Ministry of Health Department of Pharmaceuticals and Health Products (Infarmed, 2022; Ministerio de Sanidad, 2022). In the case of Italy, data were extracted from annual reports published by the AIFA Medicines Utilization Centre (Osmed). More information on data collection and reporting within these databases is available in the Supplementary Materials (OsMed, 2010, 2011, 2012, 2013, 2014, 2015, 2016, 2017, 2019, 2020; AIFA, 2022).

#### 2.1.1 Evolution of consumption volumes

##### 2.1.1.1 Cross-country comparison of volume trends within the TNF-alfa inhibitors class

**FIGURE 1:**
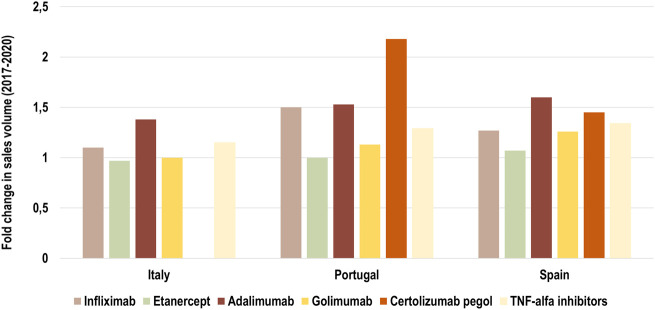
Fold change in consumption volume for all marketed TNF-alfa inhibitor molecules in Italy, Portugal and Spain in the period 2017–2020. Data are plotted for each TNF-alfa inhibitor molecule, and for the combination of all marketed TNF-alfa inhibitor molecules: infliximab, etanercept, adalimumab, golimumab, certolizumab pegol (see light yellow bar). The evolution in consumption volume for certolizumab pegol is not represented in the case of Italy, due to missing data.

**FIGURE 2:**
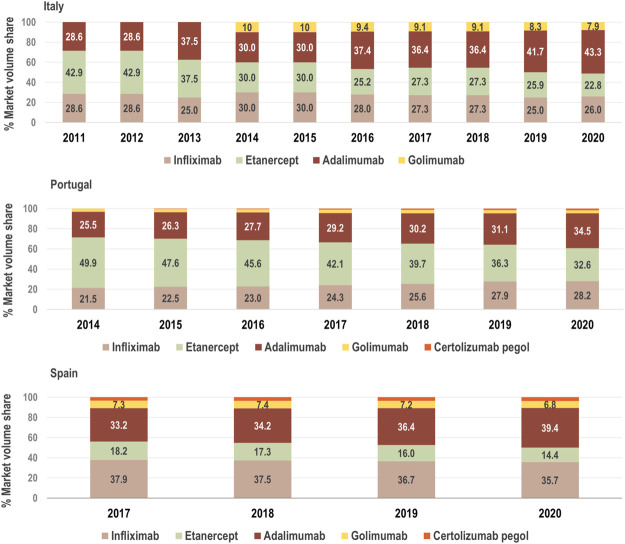
Composition of the market for TNF-alfa inhibitors in terms of active molecules from 2011, 2014 and 2017 to 2020 in Italy, Portugal and Spain, respectively. The consumption volume of each molecule is expressed as a share (%) of the total volume of the TNF-alfa inhibitors market. See Supplementary Table S1 for information on the availability of TNF-alfa inhibitor biosimilars in Italian, Portuguese, and Spanish markets. The % market volume share for certolizumab pegol is not represented in the case of Italy due to missing data.

We examined the growth of the market (fold change in consumption volume) for TNF-alfa inhibitors in the years 2017–2020, and we compared the evolution of consumption volume for each marketed TNF-alfa inhibitor product, and for the whole class in the three countries of study (see Figure 1). We then analyzed shifts in drug utilization across the TNF-alfa inhibitors class for Italy (2011–2020), Portugal (2014–2020) and Spain (2017–2020). For this analysis, we expressed the composition of the market for each TNF-alfa inhibitor molecule as a share (%) of the total volume of TNF-alfa inhibitors (see Figure 2). The timeframe chosen for the analysis allowed to study market dynamics pre- and post-TNF-alfa inhibitor biosimilars availability.

##### 2.1.1.2 Shifts in drug utilization in the rheumatology area

Data provided by The Rheumatic Diseases Portuguese Register (Reuma.pt) (Canhão et al., 2022) allowed us to focus the analysis of shifts in drug utilization on a specific therapeutic domain (rheumatology), and to extend it beyond TNF-alfa inhibitor molecules to competing biologic disease-modifying molecules (Canhão et al., 2022). We represented the composition of the biologics market for the indications ankylosing spondylitis, psoriatic arthritis, and rheumatoid arthritis (see Figures 3A–C). Per indication, we described the evolution (2011–2017) in the number of patients receiving each biologic molecule as a share (%) of the total volume of patients receiving active treatment with biologic disease-modifying molecules.

**FIGURE 3A:**
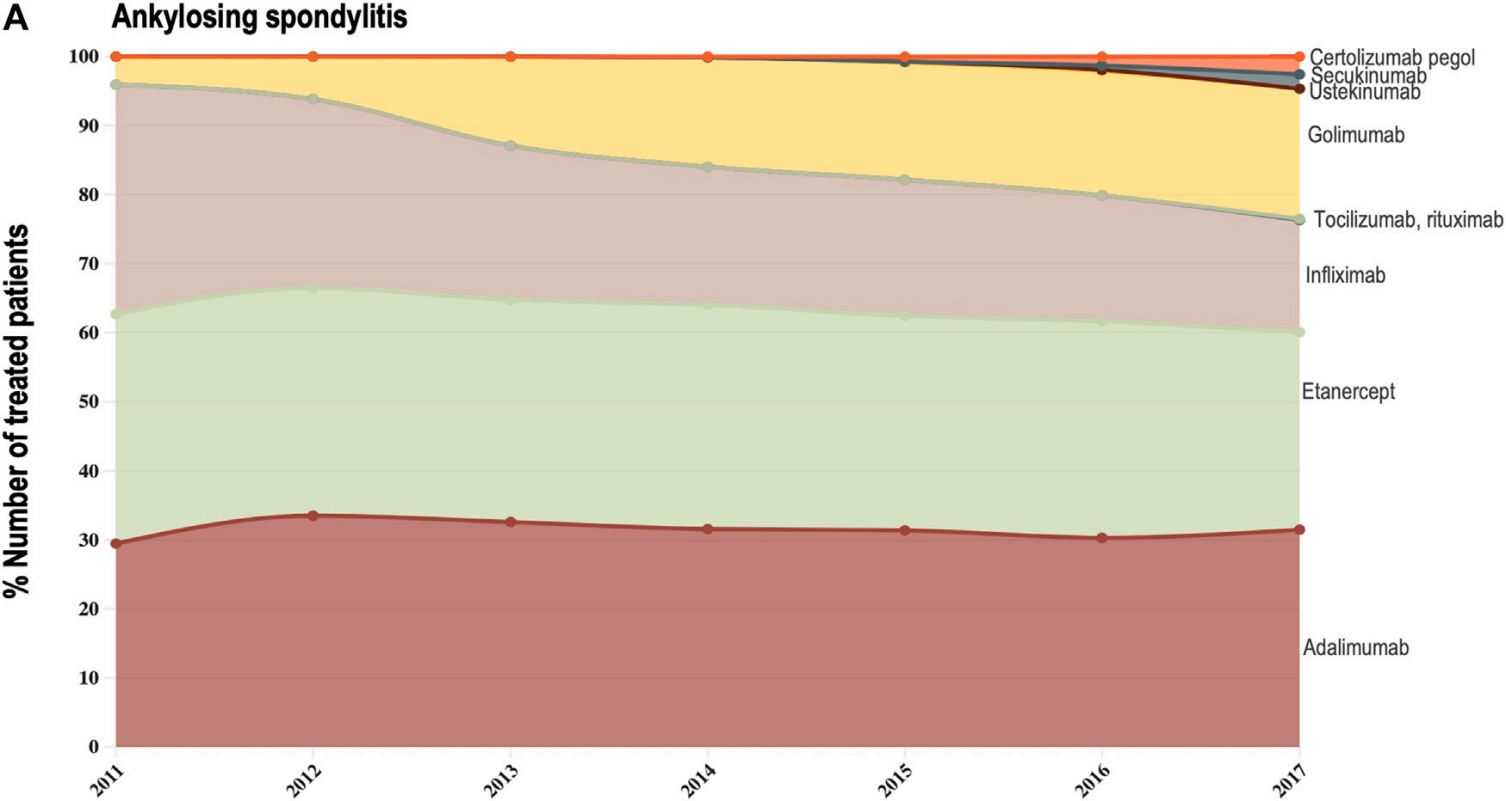
**(A)** Evolution in the composition of the biologics market for the indication ankylosing spondylitis. This figure describes the evolution in the number of patients receiving each molecule as a share (%) of the total number of patients receiving active treatment with biologic disease-modifying molecules.

**FIGURE 3B:**
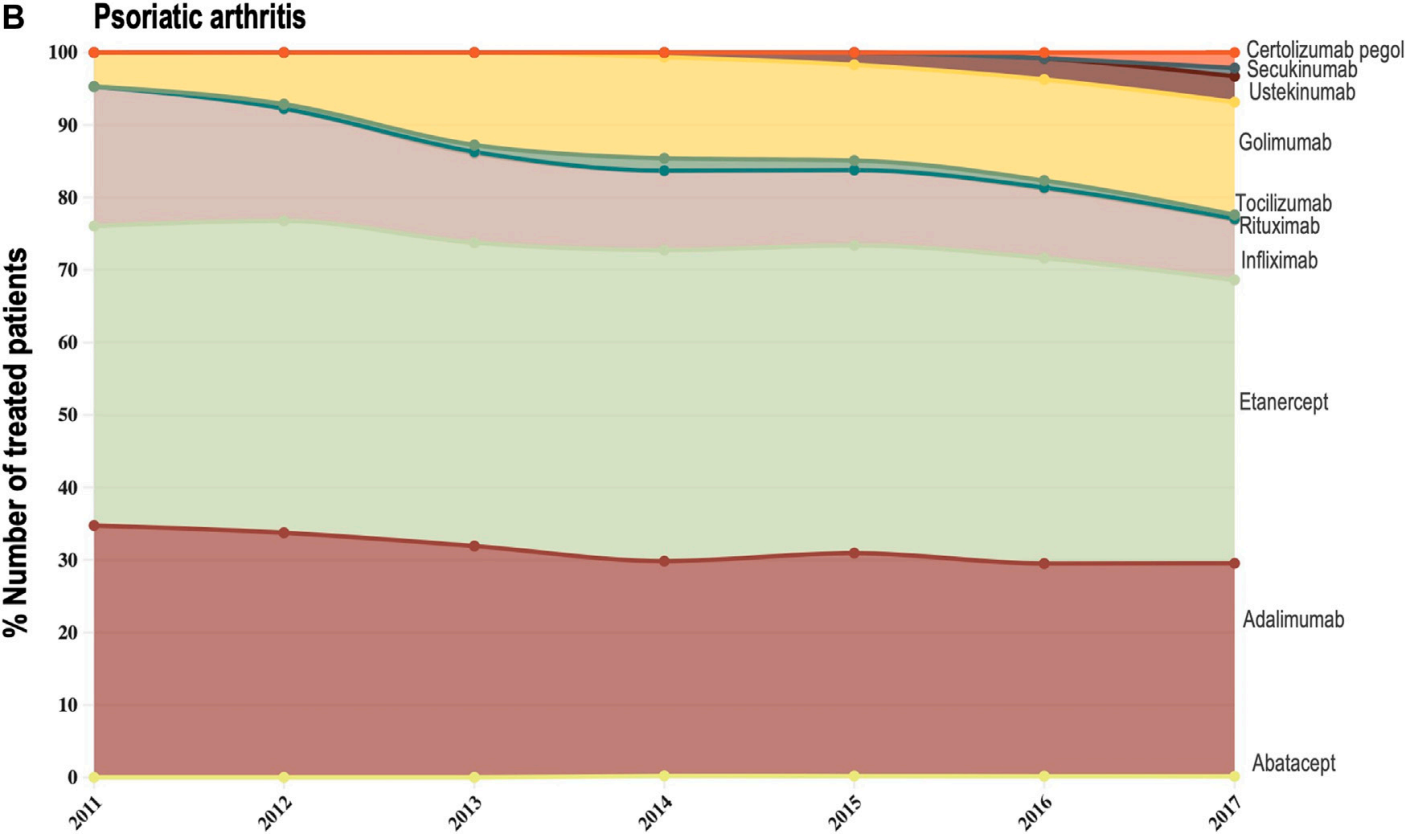
**(B)** Evolution in the composition of the biologics market for the indication psoriatic arthritis. This figure describes the evolution in the number of patients receiving each molecule as a share (%) of the total number of patients receiving active treatment with biologic disease-modifying molecules.

**FIGURE 3C:**
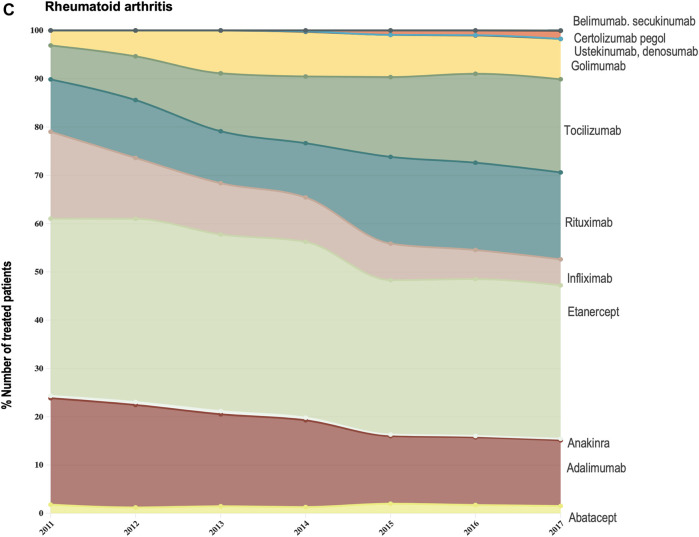
**(C)** Evolution in the composition of the biologics market for the indication rheumatoid arthritis. This figure describes the evolution in the number of patients receiving each molecule as a share (%) of the total number of patients receiving active treatment with biologic disease-modifying molecules.

#### 2.1.2 Interplay of volume and cost evolutions

We analysed the evolution in National Health Services’ costs associated with the acquisition of TNF-alfa inhibitor therapies and competing products (other biologic disease-modifying antirheumatic drugs: bDMARDs; and targeted synthetic disease-modifying antirheumatic drugs: tsDMARDs) in relation to the evolution in consumption volumes. Based on available data for the years 2018–2020 for Italy, Portugal and Spain we represented the yearly percent units change in costs in relation to the yearly percent units change in consumption volume (see Figures 4A–C). Positive percent cost and volume changes indicated an increase in costs and consumption volume in relation to the previous year. Negative percent cost and volume changes indicated a decrease in costs and consumption volume in relation to the previous year.

**FIGURE 4A:**
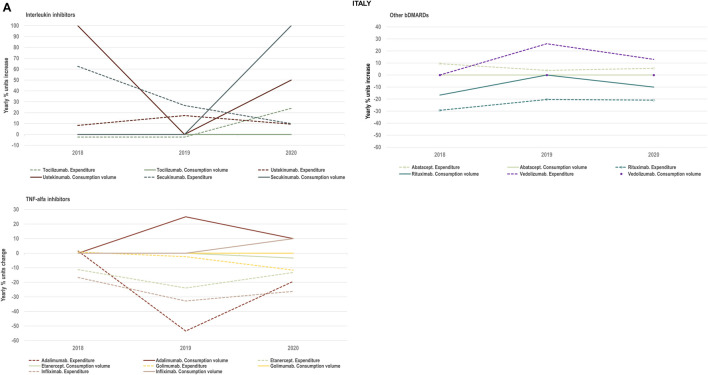
**(A)** Percent units change in costs for TNF-alfa inhibitor biologics and competing bDMARDs in relation to the percent units change in volume for these products in the period 2018–2020. Data relative to Italy.

**FIGURE 4B:**
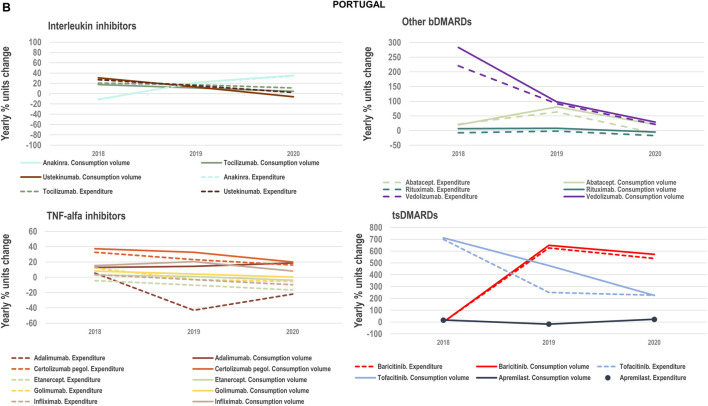
**(B)** Percent units change in costs for TNF-alfa inhibitor biologics and competing bDMARDs/tsDMARDs in relation to the percent units change in volume for these products in the period 2018–2020. Data relative to Portugal.

**FIGURE 4C:**
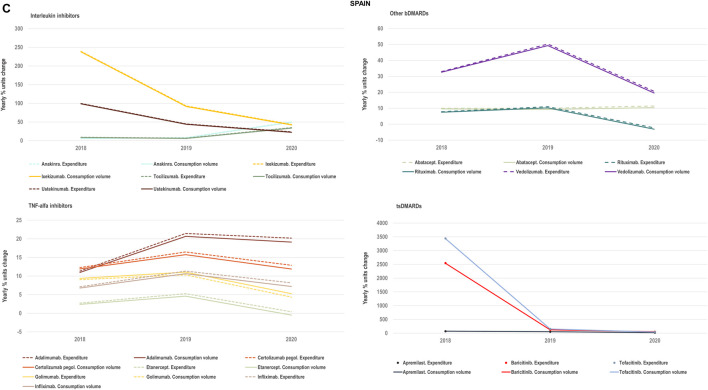
**(C)** Percent units change in costs for TNF-alfa inhibitor biologics and competing bDMARDs/tsDMARDs in relation to the percent units change in volume for these products in the period 2018–2020. Data relative to Spain.

## 3 Results

### 3.1 The market for TNF-alfa inhibitor biologics in Italy, Portugal and Spain

National Health Services’ expenditure associated to the purchase of on-patent biologic immunomodulators in Italy, Portugal and Spain has increased in the past years. The market presence of off-patent TNF-alfa inhibitor molecules and the availability of biosimilar versions for these molecules (see approval and commercialization dates in Supplementary Table S1) has been instrumental to reduce spending in the immunology area. For instance, competitive purchasing procedures that incorporate TNF-alfa inhibitor biosimilars and allow the selection of ‘best-value’ products have generated notable savings within the immunomodulators and immunosuppressants drug class. Garcia-Goñi et al. recently estimated that the market entry of TNF-alfa inhibitor biosimilars in the period 2009–2019 led to EUR 1,265M savings for the Spanish National Health Service (SNS: Servicio Nacional de Salud), and that infliximab biosimilars were the primary contributors to savings generation (García-Goñi et al., 2021). Published Portuguese SNS reports do not yet indicate the contribution of all marketed TNF-alfa inhibitor biosimilars to savings generation. However, INFARMED has estimated that if infliximab and adalimumab biosimilars would not have entered the market, SNS hospitals may have spent extra EUR 18.9M and 39M, respectively (Infarmed Notícias, 2019).

Despite the opportunity generated by biosimilars to reduce spending, previous research conducted in Italy, Portugal and Spain signaled sub-optimal market environments for off-patent biologics and biosimilars (IQVIA, 2020a; b; 2021b). This has been partly reflected in the low-uptake for TNF-alfa inhibitor biosimilars in regions where National Health Service’s costs associated to originator molecules have been higher than biosimilar costs. 2021 AIFA data showed that despite the lower cost per DDD for TNF-alfa inhibitor biosimilars in relation to the originator, the uptake for Enbrel^®^ and Humira^®^ in the regions Abruzzo, Molise and Calabria was higher than the biosimilars uptake (see Table 1). Similar situations were reported in Portugal and Spain.

**TABLE 1:** Regional data on biosimilar market shares (%) and National Health Service’s costs (SSN: Servizio Sanitario Nazionale) for infliximab-, etanercept- and adalimumab-containing products in Italy (2021).

**Infliximab (IV)**	Biosimilars Market Share (%)	Originator Cost/DDD	Biosimilars Cost/DDD	**Etanercept (SC)**	Biosimilars Market Share (%)	Originator Cost/DDD	Biosimilars Cost/DDD	**Adalimumab (SC)**	Biosimilars Market Share (%)	Originator Cost/DDD	Biosimilars Cost/DDD
**Bolzano**	100	-	3.7	**Aosta Valley**	98.8	31.5	7.4	**Aosta Valley**	100	-	3.6
**Aosta Valley**	100	-	4.0	**Bolzano**	94.7	31.5	11.2	**Piedmont**	98.2	12.8	3.6
**Sicily**	99.6	10.8	2.9	**Tuscany**	94.3	21.6	9.3	**T-ST**	96.9	20.7	5.1
**Piedmont**	99.5	10.8	3.8	**Sardinia**	92.1	23.0	20.5	**Bolzano**	97.3	20.7	5.2
**Lazio**	98.9	10.8	3.4	**T-ST**	90.8	31.5	12.0	**Sardinia**	95.7	12.8	5.2
**Tuscany**	98.6	10.8	3.7	**Sicily**	88.4	24.6	7.7	**Tuscany**	94.3	20.7	4.0
**Liguria**	97.6	10.8	5.4	**Piedmont**	87.2	31.5	7.4	**Sicily**	88.3	11.4	4.5
**Sardinia**	96.8	10.8	4.9	**ER**	86.4	31.5	8.5	**ER**	88.0	20.4	5.3
**Campania**	96.7	10.8	2.6	**Veneto**	75.6	22.5	12.3	**Veneto**	86.7	12.6	3.0
**ER**	94.4	10.9	3.2	**Basilicata**	72.6	25.2	9.3	**Lazio**	82.5	9.9	3.6
**T-ST**	93.1	10.8	5.2	**Lazio**	70.9	22.1	12.1	**Liguria**	80.4	20.7	5.2
**Veneto**	90.8	10.8	5.6	**F-VG**	69.3	28.6	11.1	**Umbria**	76.7	19.4	4.4
**F-VG**	91.8	10.8	4.0	**Liguria**	66.1	30.4	13.4	**Apulia**	65.5	16.6	5.1
**Lombardy**	89.6	10.9	2.7	**Apulia**	66.5	22.3	8.8	**F-VG**	61.8	20.1	7.1
**Marche**	89.7	10.8	6.0	**Umbria**	61.4	23.7	15.3	**Lombardy**	62.9	11.0	3.7
**Apulia**	89.6	10.8	2.8	**Lombardy**	59.1	23.2	8.5	**Marche**	62.0	12.0	5.2
**Basilicata**	88.3	10.8	3.2	**Marche**	60.4	23.7	16.5	**Campania**	59.1	20.7	3.2
**Umbria**	76.9	10.8	3.9	**Campania**	54.6	31.5	7.2	**Basilicata**	56.0	20.7	3.3
**Abruzzo**	71.4	10.8	7.9	**Abruzzo**	40.9	31.5	18.2	**Abruzzo**	43.2	17.7	5.1
**Calabria**	62.2	10.2	4.4	**Calabria**	22.4	31.5	14.2	**Molise**	28.9	17.9	5.1
**Molise**	60.0	10.8	8.5	**Molise**	13.6	31.5	18.8	**Calabria**	22.6	20.7	3.4
**Italy**	93.4	10.8	3.7	**Italy**	72.1	26.6	10.5	**Italy**	76.2	16.1	4.1

Note: Biosimilar market shares are expressed as a percentage and represent the consumption volume (DDDs) of biosimilar products, over the volume of biosimilars plus the respective originator product for each region, and for Italy (average). National Health Service’s costs per defined daily dose (DDD) of originator and biosimilar products are expressed in euros for each region, and for Italy (average) (AIFA, 2021c). Table 1 describes the evolution of average costs per DDD detected in the channel of direct purchases (NSIS). This allows to show regional differences relative to the efficiency of purchases (regional public tenders). Information on rebates after regional tender negotiations is not included.

Abbreviations: DDD, defined daily doses; cost/DDD, cost per defined daily dose; ER, Emilia-Romagna; F-VG, Friuli-Venezia Giulia; IV, intravenous; SC, subcutaneous; T-ST, Trentino-South Tyrol

In addition to detecting areas in Italy, Portugal and Spain where TNF-alfa inhibitor biosimilars uptake has been lower than for the originator, we observed high intra-country heterogeneity in biosimilars use for the molecules of study (see Table 2). After more than 6 years since TNF-alfa inhibitor biosimilars market availability, up to 87.3% and 91.1% regional variation in biosimilars uptake has been observed for Italy and Spain, respectively. This variability may be associated to the decentralized nature of healthcare competences, and to the design of biosimilar policies at the regional and local level (Caputi et al., 2016; ACSS, 2017; Brogonzoli et al., 2018; AIReF, 2020; Guidotti E et al., 2020). In Portugal, although certain competences of the health administration are delegated to the regional level, biosimilar policies have been broadly implemented at the central level. Here, the regional variability in the uptake of TNF-alfa inhibitor biosimilars has been less pronounced.

**TABLE 2:** Infliximab, etanercept and adalimumab biosimilar market shares (%) in Italy, Portugal and Spain (2020).

	Italy	Portugal	Spain
**Infliximab (IV)**			
Biosimilar market shares (%)–National average 2020	91.2	84.5	75.5
Biosimilar market shares (%)–Intra-country variability range 2020	57.4 (Molise)–100.0 (Aosta Valley)	73.9 (LVT)–99.2 (Algarve)	49.2 (Extremadura)–95.7 (Asturias)
**Etanercept (SC)**			
Biosimilar market shares (%)–National average 2020	65.8	45.1	48.7
Biosimilar market shares (%)–Intra-country variability range 2020	7.8 (Molise)–95.1 (Aosta Valley)	10.4 (Algarve)–63.8 (Center)	0 (Melilla)–91.1 (Asturias)
**Adalimumab (SC)**			
Biosimilar market shares (%)–National average 2020	64.8	33.7	42.9
Biosimilar market shares (%)–Intra-country variability range 2020	10.8 (Calabria)–96.0 (T-ST)	14.2 (LVT)–53.4 (Center)	26.5 (Melilla)–96.5 (Asturias)

Note: Biosimilar market shares are expressed as a percentage and represent the consumption volume (DDDs) of biosimilar products, over the volume of biosimilars plus the respective originator product. For the three molecules of study, we report on national average biosimilar market shares and on the intra-country maximum variability range in biosimilar market shares. We include in this table the regions with the highest and lowest biosimilars uptake (AIFA, 2020b, AIFA, 2021a; Infarmed, 2021).

Abbreviations: DDD, defined daily doses; cost/DDD, cost per defined daily dose; IV, intravenous; LVT, Lisbon and Tagus Valley; SC, subcutaneous; T-ST, Trentino-South Tyrol

### 3.2 Impact of TNF-alfa inhibitor biosimilars availability on volume trends

The consumption volume of TNF-alfa inhibitor products has increased in the past years (1.15-fold increase in Italy; 1.29-fold increase in Portugal; 1.34-fold increase in Spain). This represents an expansion in the overall access to TNF-alfa inhibitor pharmaceuticals (see Figure 1). Adalimumab- and infliximab-containing products still dominate the TNF-alfa inhibitors market in all the countries of study (see Figure 2). However, there has been an evolution in utilization trends in favor of the use of on-patent TNF-alfa inhibitors (i.e., Cimzia^®^, Simponi^®^), even after biosimilars availability.

Following TNF-alfa inhibitor biosimilars availability, the market presence of adalimumab-containing products augmented with respect to other TNF-alfa inhibitor molecules. In Italy for instance, the volume share of adalimumab increased by 5.31% in the first year or adalimumab biosimilars availability. In Portugal and Spain, a similar trend was observed, although less pronounced (See Figure 2). Conversely, the market availability of etanercept biosimilars did not lead to higher utilization of etanercept-containing products (See Figure 2; etanercept % volume share decreased by 20 points in 8 years in Italy). This has been partly due to shifts in utilization trends towards on-patent market competitors.

We characterized shifts in utilization trends for TNF-alfa inhibitors and for competing classes in Portugal, based on data provided by the Rheumatic Diseases Portuguese Registry (Canhão et al., 2022) for the indications rheumatoid arthritis (RA), Psoriatic arthritis (PsA) and ankylosing spondylitis (AS). The Rheumatic Diseases Portuguese Registry, which is the reference national-level database for rheumatic diseases, has registered an increase in the number of rheumatic patients receiving active treatment with biologics (Santos et al., 2017). The total number of patients receiving biologics treatment in 2020 was 3.1 times higher than in 2011 for RA patients, 5.5 times higher for AS patients and 5.1 times higher for PsA patients. This effectively indicates an expansion in the access to biologic treatments in the disease area.

For the three indications of study, RA, PsA and AS, we observed a shift in utilization trends from off-patent TNF-alfa inhibitors (i.e., infliximab and etanercept) towards certolizumab pegol and golimumab and towards other competing products (e.g., interleukin inhibitors, rituximab). This is despite the market entry of infliximab and etanercept biosimilars, available at lower acquisition costs. The biggest shift in drugs utilization was reported in the indication ankylosing spondylitis (See Figure 3A). In a 6-year period, the number of patients treated with off-patent biologics decreased by 21.54%. This was accompanied by a simultaneous increase in the number of patients treated with golimumab, and by a less pronounced increase in the prescription of certolizumab pegol and the IL-inhibitor products secukinumab and ustekinumab. A similar shift in drugs utilization was observed for psoriatic arthritis patients (See Figure 3B). The analysis conducted for the rheumatoid arthritis population showed that decreases in the percent number of patients treated with infliximab, etanercept and adalimumab products were accompanied by concurrent increases in the percent number of patients treated with rituximab, golimumab and tocilizumab (See Figure 3C). Overall, our analysis exhibited that the evolution of biologic treatment patterns differed per therapeutic indication, but that in general, there was a market dominance of on-patent biologics despite the availability of infliximab and etanercept biosimilars. In the case of RA patients for example, 62.8% of the patients treated with biologics in 2017 received an on-patent product.

### 3.3 Impact of TNF-alfa inhibitor biosimilars availability on spending trends

We examined the evolution in costs incurred by National Healthcare Systems to fund TNF-alfa inhibitors and competing pharmaceuticals in relation to the evolution in consumption volumes for these products. Figures 4A–C represent the percent units change in costs for TNF-alfa inhibitor biologics and competing bDMARDs/tsDMARDs relative to the percent units change in volume for these products in the period 2018–2020. In general, National Health Services’ expenses associated to infliximab- and adalimumab-containing products were lower in 2020 with respect to 2018, despite a sustained increase in consumption volume for these products. This signifies that savings were generated within the National Health Services despite expanded access to TNF-alfa inhibitor therapies.

More precisely in Italy (See Figure 4A) and Portugal (See Figure 4B), the yearly percent decrease in expenditure on adalimumab following biosimilars availability was 55% and 43%, respectively. This reduction in expenditure was observed notwithstanding a percent units increase in adalimumab utilization of 25% in Italy and of 14% in Portugal. It is to be noted that National Health Service’s expenditure associated to other molecules - namely golimumab in Italy; etanercept, golimumab and rituximab in Portugal; and rituximab in Spain- also decreased over time (See Figure 4C). However, in these cases their consumption volume remained stable or even decreased. For the rest of the molecules included in Figures 4A–C, the percent change in expenditure was positive and therefore, increased over time to a greater or lesser extent.

Data presented in Figures 4A–C show the potential of TNF-alfa inhibitors biosimilars availability to decrease National Health Services’ expenditure within the TNF-alfa inhibitors class, including the on-patent product golimumab. Expenditure decreases have however not always been accompanied by expanded access to these therapies (see the case of etanercept). This seems to be due to the shift in drugs utilization towards other competing on-patent molecules, whose National Health Services’-associated expenditure has increased over time.

## 4 Discussion

Immunosuppressant and immunomodulating agents belong to the highest expenditure category of hospital-use pharmaceuticals in Italy, Portugal and Spain. Within this category, patent-covered biologic and targeted synthetic disease-modifying agents account for most of the expenditure (Ministerio de Sanidad, 2019b; SPMS, 2019; AIFA, 2020c; d). It has been a goal of the administration of the National Health Services in these countries to control pharmaceutical spending by fostering optimal market environments for off-patent biologics and biosimilar medicines. This has been reflected in the issuance of plans at the national/regional level to increase the utilization of these medicines (ACSS, 2017; AIFA, 2018; Ministerio de Sanidad, 2019a,; AIFA, 2021b).

The availability of TNF-alfa inhibitor biosimilars in Southern European markets has supported the earlier and expanded access to these therapies (Smolen et al., 2017; Smolen et al., 2020). This has been reflected in our analysis. However, despite the dominance of infliximab- and adalimumab-containing products on the TNF-alfa inhibitors market, the percent volume share of etanercept has decreased in the past years, even after biosimilars availability. A combination of factors can explain this observation: 1) the more limited indications profile of etanercept in relation to infliximab and adalimumab (EMA, 2022); 2) the more difficult adoption/switch to subcutaneous biosimilar formulations in relation to intravenous formulations; 3) the observed drug utilization shifts from off-patent TNF-alfa inhibitors towards second-generation products (Cimzia^®^ and Simponi^®^), and IL-inhibitors in the rheumatology area.

Even though costs per DDD for infliximab, etanercept and adalimumab biosimilars have been generally lower than originator costs in the studied markets, we detected 1) high intra-country heterogeneity in biosimilars uptake and 2) areas where TNF-alfa inhibitor biosimilars uptake was lower than 25%. After 6 years of biosimilars availability in the TNF-alfa inhibitors market and considering European-level uptake data, a higher biosimilars adoption could have been expected from Southern European markets (IQVIA, 2020b; IQVIA, 2021b). The heterogenous uptake of biosimilars, especially observed in Italy and Spain, has been associated with the decentralized nature of the healthcare systems’ organization (Marcianò et al., 2016; Rémuzat et al., 2017). This has led to different regional models for the purchasing and procurement of biologics, and to a plurality of policy-making centers. More research is needed to investigate drivers of biosimilars uptake in Southern European markets and to understand regional/local factors affecting biosimilars adoption with a view to propose policies that can address still unresolved barriers to biosimilars use.

Regarding savings generated following TNF-alfa inhibitor biosimilars availability, data analysed in this study suggest that cost reductions attained are notable for the molecules directly exposed to biosimilars competition (i.e., infliximab, etanercept, adalimumab). For these molecules, National Health Services’ costs in 2020 were lower than in previous years despite stable or increased consumption volume. However, the extent of cost savings is rather limited for on-patent molecules within the drug class (i.e., Cimzia^®^ and Simponi^®^). This confirms the notion that the scope of the competition between different active molecules is limited and showcases the need for strategies that can fully leverage biosimilars competition (AGCM, 2019). Our research also shows that price dynamics for competing molecules outside of the TNF-alfa inhibitors class are not altered. The limitations in the extent of price reductions attained after biosimilars availability can be understood in the context of 1) purchasing procedures that do not stimulate competition across product classes, and 2) the natural evolution of care standards for patients diagnosed with chronic immune-mediated inflammatory conditions. In this line, our analysis indicated that shifts in drugs utilization towards on-patent immunomodulators occurred after loss of exclusivities for TNF-alfa inhibitors, notwithstanding higher acquisition costs. As indicated by Vandenplas et al., there can be multiple determinants of prescribing decisions (Vandenplas et al., 2022). Shifts in prescribing from off-patent TNF-alfa inhibitors towards higher-cost second and third generation on-patent products could be partly due to the greater availability of dedicated physician education and patient support programs for these products. Further, aspects such as the lack of prescribers’/patients’ trust on biosimilars could have motivated these shifts. More research is needed to investigate drivers of prescribers’ decisions in specific scenarios.

Based on our research, it becomes relevant to implement policies that support the willingness of biosimilar manufacturers to keep investing in the long-term development of these medicines. This is to ensure the sustainable development of biosimilar candidates for drug classes that do not yet face biosimilar competition. Market forecasts indicate that the next wave of biosimilar approvals in the immunology area would lead to biosimilar candidates for RoActemra^®^/Actemra^®^ (tocilizumab), Cimzia^®^ (certolizumab pegol), Simponi^®^ (golimumab) and Stelara^®^ (ustekinumab). Although the main patents for Actemra^®^ and Cimzia^®^ already expired, biosimilar candidates for these molecules are not yet being evaluated for approval by the EMA. The fact that there are multiple biosimilar candidates in development for tocilizumab and ustekinumab is encouraging. However, it is unclear whether the market entry of these products will result in strong competitive pressure within the category of immunomodulator/immunosuppressant drugs. Also, estimates indicate that various originator biologic immunosupressants/immunomodulators whose patents are set to expire in the coming years, may not face biosimilar competition in the short-term (BR&R, 2020; Akram et al., 2021; BR&R, 2021). Future research projects would benefit from studying the combined impact of the market availability of competing biosimilars (e.g., TNF-alfa/IL- inhibitors), and JAK inhibitor generics on savings and treatment access generation in immunology.

### 4.1 Added value of the study and limitations

Previous studies conducted by our research group have focused on capturing the effect of TNF-alfa inhibitor biosimilar competition within the TNF-alfa inhibitors class (Moorkens et al., 2019a; Moorkens et al., 2019b; Moorkens et al., 2020; Moorkens et al., 2021). However, biosimilars availability may also influence market dynamics for competing products in other therapeutic classes. By capturing the evolution of consumption volume and National Health Services’ expenditure data after TNF-alfa inhibitor biosimilars market entry for competing immunomodulator drugs, the current study provides a more comprehensive view on the potential benefits of biosimilar competition. Having conducted this analysis constitutes an added value with respect to previously published research. Further, to accurately represent shifts in drugs utilization before and after TNF-alfa inhibitor biosimilars availability, we have focused our analysis on a specific setting (Portuguese hospital setting) and a concrete disease area (rheumatology). This case study constitutes an interesting addition to the current body of knowledge regarding the evolution of drug utilization trends in immunology.

Despite the added value of this research, some limitations need to be acknowledged. These pertain to data availability and different standards for data reporting in the countries of study and have determined the scope and timeframe chosen for our analyses. The analysis of market dynamics has been based on the availability of data from National Databases in Italy, Portugal and Spain. It is to be noted that the data analysed do not represent the whole TNF-alfa inhibitors market in the countries of study. However, TNF-alfa inhibitors consumption volume and acquisition cost evaluations for publicly managed hospitals within the National Health Systems network are accurately represented (See Supplementary Materials for information on data sources).

Regarding the inclusion of molecules in the analysis, we aimed to incorporate all marketed TNF-alfa inhibitors and all relevant competitors. Although the list of molecules selected is not complete, we managed to include most relevant market competitors (*n* = 13, abatacept, anakinra, apremilast, baricitinib, certolizumab pegol, golimumab, ixekizumab, rituximab, secukinumab, tocilizumab, tofacitinib, ustekinumab, vedolizumab). It is noteworthy that we studied molecules with different indications and formulations profiles. Consumption trends were compared for a broad range of subcutaneous formulations and for a more limited range of intravenous formulations. Unlike for intravenous formulations, dosage of subcutaneous formulations can be modulated by patients. The effect of dose modulation on consumption trends has not been discussed within this manuscript. Furter, differences in consumption trends for the molecules studied could be partly due to the different indications profile of these molecules (e.g., limited vs. broad range of indications; indications granted at different moments in time). Also, registered indications for originator products do not necessarily coincide with registered indications for the respective biosimilars. These differences increase the complexity of analysis of volume trends evolution. Volume trends can in fact reflect prescribers’ lack of familiarity with newly granted indications for specific molecules. Based on our data, it was not possible to analyze these factors.

Due to data limitations as well, we only evaluated shifts in drugs utilization (2011–2017) in the rheumatology area in Portugal. Here, it would have been optimal to provide data at least up to 2020 (after the market entry of adalimumab biosimilars). However, this was not possible due to missing information from 2017 onwards. Future research would benefit from expanding our Portuguese case study on drug utilization shifts in rheumatology to Italy and Spain. Future research would also benefit from analyzing the interplay of volume and cost evolutions for TNF-alfa inhibitors and competing products at the regional level in the countries of study. Due to procurement activities organized at the regional level in these countries, these dynamics have varied regionally.

## 5 Conclusion

Our analysis showed that the availability of TNF-alfa inhibitor biosimilars in Southern-European markets led to lowered National Health Services’ costs within the drug class, even after an increased consumption volume for these therapies. However, the potential of marketed TNF-alfa inhibitor biosimilars to generate price competition outside their drug class has been limited. This limitation becomes especially relevant in the context of observed drug utilization shifts towards on-patent and less-affordable therapies, even after loss of exclusivities for Remicade^®^, Enbrel^®^ and Humira^®^. In light of these data, the need for policies that do not only seek higher utilization of marketed ‘best-value’ biologics, but that also support the sustainable development of new biosimilar molecules is highlighted.

## Data Availability

The datasets generated and/or analyzed during the current study are available from the corresponding author on reasonable request.

## Supplementary material

Supplementary Table S1 List of EMA-approved TNF-alfa inhibitor pharmaceuticals. For the active molecules infliximab, etanercept, adalimumab, certolizumab pegol and golimumab we provide a list of EMA-approved indications and administration routes and the date of marketing approval of the originator and biosimilar products (if available). For infliximab, etanercept and adalimumab we specify the year of availability of the first biosimilar product in Italian, Portuguese and Spanish markets.

Supplementary Table S2 List of biologic and targeted synthetic disease-modifying pharmaceuticals used in the treatment of immune-mediated inflammatory conditions and that are market competitors for TNF-alfa inhibitors. The inclusion of molecules in this list has been based on the review of country-specific(Asociación Española de Gastroenterología; Buch et al., 2011; Sociedad Española de Reumatología, 2014; Torres et al., 2016; Duarte et al., 2017; Gisondi et al., 2017; Lamb et al., 2019; Torres et al., 2020b; AEG. Asociación Española de Gastroenterología, 2021; FINISTERRA, 2021; NICE. National Institute for Health and Care Excellence, 2021) and European guidelines (Nast et al., 2017; Smolen et al., 2020; Torres et al., 2020a; Sriranganathan et al., 2021) published by rheumatology, gastroenterology, and dermatology associations.
