# Supplementary material for: Evaluating the benefits of TNF-alfa inhibitor biosimilar competition on off-patent and on-patent drug markets: A Southern European analysis
Source: Front Pharmacol. 2022 Dec 16;13:1031910. doi: 10.3389/fphar.2022.1031910 (PMC9802634; doi:10.3389/fphar.2022.1031910)
Supplement: Supplementary file 1 [file DataSheet1.pdf]

Supplementary Material

1 Supplementary Tables

Supplementary Table 1. List of EMA-approved TNF-alfa inhibitor pharmaceuticals. For the active molecules infliximab, etanercept, adalimumab, certolizumab pegol and golimumab we provide a list of EMA-approved indications and administration routes and the date of marketing approval of the originator and biosimilar products (if available). For infliximab, etanercept and adalimumab we specify the year of availability of the first biosimilar product in Italian, Portuguese and Spanish markets.

**Molecule INN**<br><br>**Structure type**	**EMA approved indications**	**Originator product**<br><br>**Name/date EMA marketing approval**	**Originator product**<br><br>**Administration routes** (for approved indications)	**Biosimilar products**<br><br>**Name/date EMA marketing approval**	**Biosimilar products**<br><br>**Administration routes** (for approved indications)	**Biosimilar products**<br><br>**Market availability** (1st approved biosimilar molecule)
**Infliximab**<br>Chimeric mouse/human anti-TNF-alfa monoclonal IgG1 antibody	-Rheumatoid arthritis (RA)<br>-Psoriatic arthritis (PsA)<br>-Ankylosing spondylitis (AS)<br>-Adult plaque psoriasis (Ps)<br>-Adult Crohn's disease (CD), paediatric CD<br>-Adult ulcerative colitis (UC), paediatric UC	**Remicade®**<br>13/08/1999	Intravenous (IV)<br><br>Remicade® does not have an authorized subcutaneous (SC) formulation	**Inflectra®**<br>10/09/2013<br>**Remsima®**<br>10/09/2013<br>19/09/2019 (extension of marketing authorization, SC formulations for RA)<br>**Flixabi®**<br>26/05/2016<br>**Zessly®**<br>18/05/2018	**Inflectra®** - IV<br>**Remsima®**<br>-IV (for all the indications included in column 2)<br>-SC (for RA)<br>**Flixabi®** - IV<br>**Zessly®** - IV	**Italy:** 02/2015<br>**Spain:** marketing authorization 03/2014<br>First sales registered in 2015<br>(Agencia Española de Medicamentos y Productos Sanitarios and CIMA)<br><br>**Portugal:** marketing authorization 10/2013<br>First sales registered in 2014 (data for the years 2013-2015 are not available within the Infarmed database) (Ana S. Freitas)
**Etanercept**<br>Extracellular domain of TNF receptor 2/IgG1-Fc fusion protein	-RA<br>-PsA<br>-AS<br>-Adult Ps, paediatric Ps<br>-Juvenile idiopathic arthritis (JIA)	**Enbrel®**<br>02/02/2000	SC	**Benepali®**<br>25/04/2016<br>**Erelzi®**<br>23/06/2016<br>**Nepexto®**<br>20/05/2020	SC	**Italy:** 10/2016<br>**Spain:** marketing authorization 02/2016<br>First sales registered in 2016<br>**Portugal**: marketing authorization 06/2016<br>First sales reported by Infarmed in September 2017
**Adalimumab**<br>Fully humanized monoclonal IgG1 antibody	-RA<br>-PsA<br>-AS<br>-Adult Ps, paediatric Ps<br>-JIA<br>-Adult CD, paediatric CD<br>-Adult UC, paediatric UC<br>-Hidradenitis suppurativa<br>-Adult uveitis, paediatric uveitis	**Humira®**<br>08/09/2003	SC	**Amgevita®**<br>21/03/2017<br>**Imraldi®**<br>24/08/2017<br>**Hyrimoz®**<br>26/07/2018<br>**Hefiya®**<br>26/07/2018<br>**Idacio®**<br>02/04/2019<br>**Amsparity®**<br>13/02/2020<br>**Yuflyma®**<br>11/02/2021<br>**Libmyris®**<br>12/11/2021<br>**Hukyndra®**<br>15/11/2021	SC	**Italy:** 10/2018<br>**Spain:** marketing authorization 04/2018<br>First sales registered in 2018<br>**Portugal:** First sales reported by Infarmed in November 2018
**Certolizumab pegol**<br>PEGylated anti-TNF Fab' fragment	-RA<br>-PsA<br>-AS<br>-Adult Ps	**Cimzia®**<br>01/10/2009	SC	Not available	N/A	N/A
**Golimumab**<br>Fully humanized monoclonal IgG1 antibody	-RA<br>-PsA<br>-AS<br>-Adult UC	**Simponi®**<br>01/10/2009	IV and SC	Not available	N/A	N/A

Information sources: ((EMA); Agencia Española de Medicamentos y Productos Sanitarios and CIMA)
Abbreviations: AS: Ankylosing spondylitis; **CD:** Crohn's disease**; EMA:** European Medicines Agency; **Fab':** Fragment antigen-binding region; **Fc:** Fragment crystallizable region; **IgG:** Immunoglobulin G; **INN:** International nonproprietary name; **IV:** intravenous; **JIA:** Juvenile Idiopathic Arthritis; **RA:** Rheumatoid arthritis; **SC:** subcutaneous; **TNF:** Tumor Necrosis Factor; **PsA:** Psoriatic arthritis; **Ps:** Plaque psoriasis; **UC:** Ulcerative Colitis.

Supplementary Table 2. List of biologic and targeted synthetic disease-modifying pharmaceuticals used in the treatment of immune-mediated inflammatory conditions and that are market competitors for TNF-alfa inhibitors. The inclusion of molecules in this list has been based on the review of country-specific(AEG; Buch et al., 2011; SER, 2014; Torres et al., 2016; Duarte et al., 2017; Gisondi et al., 2017; Lamb et al., 2019; Torres et al., 2020b; AEG, 2021; FINISTERRA, 2021; NICE. National Institute for Health and Care Excellence, 2021) and European guidelines (Nast et al., 2017; Smolen et al., 2020; Torres et al., 2020a; Sriranganathan et al., 2021) published by rheumatology, gastroenterology, and dermatology associations.

**Molecule INN**<br><br>**Structure type**<br><br>**Molecule target**	**EMA approved indications**	**Originator product**<br><br>**Name/date EMA marketing approval**	**Originator product**<br><br>**Administration routes**<br>**(for approved indications)**	**Biosimilar/generic product**<br><br>**Name/date EMA marketing approval**	**Biosimilar products**<br><br>**Administration routes**<br>**(for approved indications)**	**Biosimilar products**<br><br>**Market availability**<br>**(1st biosimilar molecule)**
**Abatacept**<br>-Combined extracellular CTLA4 and IgG1 Fc domains<br>-Anti-CD80, anti-CD86. It blocks the activation of T-cell Ab4Ig	-Rheumatoid arthritis (RA)<br>-Psoriatic arthritis (PsA)<br>-Polyarticular juvenile idiopathic arthritis (pJIA)	**Orencia®**<br>21/05/2007	Intravenous (IV)<br>Subcutaneous (SC)	Not available	N/A	N/A
**Anakinra**<br>-Recombinant human IL-1 receptor antagonist<br>-Anti-IL1	-RA<br>-COVID-19<br>-Periodic fever syndromes<br>-Still's Disease	**Kineret®**<br>08/03/2002	SC	Not available	N/A	N/A
**Apremilast**<br>-Targeted synthetic disease-modifying antirheumatic drug (tsDMARD)<br>-Phosphodiesterase 4 (PDE4) inhibitor	-PsA<br>-Psoriasis	**Otezla®**<br>15/01/2015	Oral	Not available	N/A	N/A
**Baricitinib**<br>-tsDMARD<br>-Janus Kinase (JAK) 1 and 2 inhibitor	-RA<br>-Atopic dermatitis	**Olumiant®**<br>13/02/2017	Oral	Not available	N/A	N/A
**Belimumab**<br>-Human recombinant IgG1 λ mAb<br>-B-lymphocyte stimulator-specific inhibitor	-Systemic lupus erythematosus (SLE)	**Benlysta®**<br>13/07/2011	IV<br>SC	Not available	N/A	N/A
**Brodalumab**<br>-Anti-IL17	-Plaque psoriasis	**Kyntheum®**<br>17/07/2017	SC	Not available	N/A	N/A
**Canakinumab**<br>-Human recombinant IgG1/k mAb<br>-Anti-IL1beta	-sJIA<br>-Gouty arthritis<br>-Periodic fever syndromes	**Ilaris®**<br>23/10/2009	SC	Not available	N/A	N/A
**Filgotinib**<br>-tsDMARD<br>-Janus Kinase (JAK) 1 and 2 inhibitors	-RA<br>-UC	**Jyseleca®**<br>24/09/2020	Oral	Not available	N/A	N/A
**Guselkumab**<br>-Human IgG1 λ mAb<br>-Anti-IL23	-Plaque psoriasis	**Tremfya®**<br>10/11/2017	SC	Not available	N/A	N/A
**Ixekizumab**<br>-Humanised IgG4 mAb<br>-Anti-IL7A	-AS<br>-PsA<br>-Plaque psoriasis	**Taltz®**<br>25/04/2016	SC	Not available	N/A	N/A
**Risankizumab**<br>-Humanized IgG1 mAb<br>-Anti-IL23	-PsA<br>-Plaque psoriasis	**Skyrizi®**<br>26/04/2019	SC	Not available	N/A	N/A
**Rituximab**<br>-Chimeric murine/human monoclonal IgG1k antibody<br>-Anti-CD20	-RA<br>-Non-Hodgkin's lymphoma (NHL)<br>-Chronic Lymphocytic leukemia (CLL)<br>-Granulomatosis with polyangiitis and microscopic polyangiitis<br>-Pemphigus vulgaris (PV)	**Mabthera®**<br>02/06/1998	IV	**Riximyo®**<br>15/06/2017<br>**Truxima®**<br>17/02/ 2017<br>**Rixathon®**<br>13/07/2017<br>**Blitzima®**<br>13/07/2017<br>**Ruxience®**<br>01/04/2020	IV<br><br>IV<br><br>IV<br><br>IV<br><br>IV	**Italy:** 07/2017<br>**Spain:** 04/2017<br>**Portugal:** 07/2017
**Sarilumab**<br>-Human IgG1 mAb<br>-Anti-IL6	-RA	**Kevzara®**<br>**23/06/2017**	SC	Not available	N/A	N/A
**Secukinumab**<br>-Human IgG1 k mAb<br>-Anti IL-17A	-AS<br>-PsA<br>-Plaque psoriasis	**Cosentyx®**<br>14/01/2015	SC	Not available	N/A	N/A
**Tildrakizumab**<br>-Humanized IgG1 k mAb<br>-Anti-IL23	-Plaque psoriasis	**Ilumetri®**<br>17/09/2018	SC	Not available	N/A	N/A
**Tocilizumab**<br>-Humanised mAb (IgG1T)<br>-Anti-IL6	-RA<br>-systemic juvenile idiopathic arthritis (sJIA)<br>-pJIA<br>-COVID-19<br>-Giant cell arteritis<br>-Cytokine release syndrome	**RoActemra®**<br>15/01/2009	IV<br>SC	Not available	N/A	N/A
**Tofacitinib**<br>-tsDMARD<br>-Janus Kinase (JAK) 1 and 3 inhibitors	-RA<br>-AS<br>-PsA<br>-pJIA<br>-UC	**Xeljanz®**<br>22/03/2017	Oral	Not available	N/A	N/A
**Upadacitinib**<br>-tsDMARD<br>-Janus Kinase (JAK) 1 selective inhibitor	-RA<br>-AS<br>-PsA<br>-Atopic dermatitis	**Rinvoq®**<br>16/12/2019	Oral	Not available	N/A	N/A
**Ustekinumab**<br>-Human IgG1k mAb<br>-Anti IL-12 and IL-23	-PsA<br>-Adult, paediatric plaque psoriasis<br>-CD<br>-UC	**Stelara®**<br>15/01/2009	IV<br>SC	Not available	N/A	N/A
**Vedolizumab**<br>-Recombinant humanized IgG1 mAb<br>-Alfa-4-beta-7 integrin blocker	-CD<br>-UC<br>-Pouchitis	**Entyvio®**<br>22/05/2014	IV<br>SC	Not available	N/A	N/A

Information sources: ((EMA); Agencia Española de Medicamentos y Productos Sanitarios and CIMA)
Table lines in orange correspond to non-biologic products (e.g., targeted synthetic disease-modifying antirheumatic drugs)

3. Data sources

The following data sources have been consulted to gather consumption volume and NHS expenditure data for TNF-alfa inhibitors and competing immunomodulators.

Italy

We have consulted reports (2011-2021) emitted by the Italian Medicines Agency (AIFA) Medicines Utilization Center (OsMeD) on the yearly use of medicines. These reports are publicly available, either in Italian or English (depending on the year) and provide a national- and regional-level overview on consumption and expenditure data (OsMed, 2001-2022; 2017). Consumption volume data have generally been expressed as defined daily doses (DDD) per 1000 inhabitants per day or as DDD per capita per day. NHS expenditure has been expressed in euros as per capita expenditure.

Data on biosimilar market shares (expressed as a percentage; represent the consumption volume of biosimilar products, over the volume of biosimilars plus the respective originator molecule) have been published within AIFA reports on the monitorization and consumption of biosimilars (data are available at the regional level since 2019)(AIFA, 2019-2022).

Portugal

The Portuguese National Authority of Medicines and Health Products (Infarmed) publishes monthly reports on the consumption and expenditure associated to high-use/high-cost medicines in the ambulatory and the hospital setting. These reports are publicly available (Infarmed, 2007-2022). However, data are not reported systematically for every product purchased by the NHS within a therapeutic class. For our analyses, data from the above-mentioned reports were complemented with consumption volume/expenditure data provided by Infarmed (for publicly managed hospitals in the regions Alentejo, Algarve, Centro, Lisboa e Vale do Tejo, Norte) for the molecules: abatacept, adalimumab, anakinra, apremilast, baricitinib, brodalumab, certolizumab pegol, etanercept, golimumab, guselkumab, infliximab, ixekizumab, rituximab, tocilizumab, tofacitinib, ustekinumab, vedolizumab. Consumption volume data have been expressed in number of units (defined by the *Código Hospitalar Nacional de Medicamentos*; tablets, capsules, pens, injectable solutions, etc.). NHS expenditure has been expressed in euros as yearly expenditure for the total number of units purchased by public NHS hospitals. Data on biosimilar market shares (expressed as a percentage; represent the consumption volume of biosimilar products, over the volume of biosimilars plus the respective originator molecule) have been published within the Infarmed website *'Medicamentos biossimilares'(SNS, 2021).*

For the analysis of shifts in drug utilization in the rheumatology area in Portugal, we analysed data published in reports emitted by The Rheumatic Diseases Portuguese Register (Reuma.pt) from 2011 to 2017 (Reuma.pt, 2011-2021). Full reports from 2018 onwards were not publicly available. Reuma.pt is a patient registry developed by the Portuguese Society of Rheumatology, gathering data from 77 care centers actively contributing to the recruitment and follow-up of rheumatic patients (rheumatoid arthritis, n=6218; psoriatic arthritis, n=1498; spondyloarthritis, n=2529; juvenile idiopathic arthritis, n=1561; autoinflammatory syndromes, n=122; systematic lupus erythematosus, n=1718; systematic sclerosis, n=180; vasculitis, n=221) (Santos et al., 2017). These reports provide data about the number of patients per indication receiving treatment with biologic disease-modifying agents (data per molecule are provided). In our analysis, for the indications ankylosing spondylitis, psoriatic arthritis

and rheumatoid arthritis, we have described the evolution (2011-2017) in the number of patients receiving each biologic molecule (i.e., abatacept, adalimumab, anakinra, belimumab, certolizumab pegol, denosumab, etanercept, golimumab, infliximab, rituximab, secukinumab, tocilizumab, ustekinumab) as a share (%) of the total volume of patients receiving active treatment with biologic disease-modifying agents.

Spain

We contacted the Department of Pharmaceuticals and Health Products within the Spanish Ministry of Health for information about consumption volume and NHS expenditure for TNF-alfa inhibitors and competing immunomodulators. We received data for the molecules: abatacept, adalimumab, anakinra, apremilast, baricitinib, brodalumab, certolizumab pegol, etanercept, filgotinib, golimumab, guselkumab, infliximab, ixekizumab, risankizumab, rituximab, sarilumab, tildrakizumab, tocilizumab, tofacitinib, upadacitinib, ustekinumab, vedolizumab.

Consumption volume data have been expressed in DDD per 1000 inhabitants per day. NHS expenditure has been expressed in euros as yearly expenditure for the total number of units purchased by public NHS hospitals. The ex-factory price has been discounted according to the specifications of the Decree Law 9/2010, 20th May (RDL 9/2010, 20 mayo) (BOE, 2010).

Data on biosimilar market shares (expressed as a percentage; represent the consumption volume of biosimilar products, over the volume of biosimilars plus the respective originator product) have been provided upon request by the Spanish Ministry of Health, Department of Pharmaceuticals and Health Products (national-level data) and the Spanish Association of Biosimilar Medicines (BioSim; national and regional-level data)

4. References

(EMA), E.M.A. *Assessment Report on extension(s) of marketing authorisation. Remsima* [Online]. Available: https://www.ema.europa.eu/en/documents/assessment-report/remsima-epar-public-assessment-report_en.pdf [Accessed].

AEG "Documento de actualización de la Guía de Práctica Clínica sobre el síndrome del instestino irritable".).

AEG (2021). *Guías de Práctica Clínica y Documents de Posicionamiento de la AEG* [Online]. Available: https://www.aegastro.es/p/documentos-posicionamiento-aeg/ [Accessed].

Agencia Española de Medicamentos y Productos Sanitarios, and CIMA. Available: https://cima.aemps.es/cima/publico/home.html [Accessed].

AIFA (2019-2022). Monitoraggio consumi e spesa biosimilari.

Ana S. Freitas Análise do Impacto Económico dos Medicamentos Biossimilares na Despensa do Serviço Nacional de Saúde. *Facultade de Economia. Universidade Do Porto*.

BOE, L.C. (2010). Real Decreto-LEY 8/2010, de 20 de mayo, por el que se adoptan medidas extraordinarias para la reducción del déficit público.

Buch, M.H., Smolen, J.S., Betteridge, N., Breedveld, F.C., Burmester, G., Dörner, T., et al. (2011). Updated consensus statement on the use of rituximab in patients with rheumatoid arthritis. *Annals of the Rheumatic Diseases* 70(6)**,** 909. doi: 10.1136/ard.2010.144998.

Duarte, C., Sousa-Neves, J., Águeda, A., Ribeiro, P., Daniel, A., Eugénio, G., et al. (2017). Portuguese Recommendations for the use of biological therapies in patients with rheumatoid arthritis- 2016 update. *Acta Reumatol Port* 42(2)(Apr-Jun)**,** 112-126.

FINISTERRA (2021). *Guías Clínicas. Psoriasis* [Online]. Available: https://www.fisterra.com/guias-clinicas/psoriasis/#29035 [Accessed].

Gisondi, P., Altomare, G., Ayala, F., Bardazzi, F., Bianchi, L., Chiricozzi, A., et al. (2017). Italian guidelines on the systemic treatments of moderate-to-severe plaque psoriasis. *J Eur Acad Dermatol Venereol* 31(5)**,** 774-790. doi: 10.1111/jdv.14114.

Infarmed (2007-2022). Análise de consumo de medicamentos em meio hospitalar.

Lamb, C.A., Kennedy, N.A., Raine, T., Hendy, P.A., Smith, P.J., Limdi, J.K., et al. (2019). British Society of Gastroenterology consensus guidelines on the management of inflammatory bowel disease in adults. *Gut* 68(Suppl 3)**,** s1. doi: 10.1136/gutjnl-2019-318484.

Nast, A., Spuls, P.I., van der Kraaij, G., Gisondi, P., Paul, C., Ormerod, A.D., et al. (2017). European S3-Guideline on the systemic treatment of psoriasis vulgaris - Update Apremilast and Secukinumab - EDF in cooperation with EADV and IPC. *J Eur Acad Dermatol Venereol* 31(12)**,** 1951-1963. doi: 10.1111/jdv.14454.

NICE. National Institute for Health and Care Excellence (2021). *NICE Pathways. Crohn's disease overview* [Online]. Available: https://pathways.nice.org.uk/pathways/crohns-disease#path=view%3A/pathways/crohns-disease/crohns-disease-overview.xml&content=view-index [Accessed].

OsMed (2001-2022). National report on medicines use in Italy

OsMed (2017). Report regionali sul consumo dei farmaci in Italia.

Reuma.pt (2011-2021). Relatórios.

Santos, M.J., Canhão, H., Mourão, A.F., Oliveira Ramos, F., Ponte, C., Duarte, C., et al. (2017). Reuma.pt contribution to the knowledge of immune-mediated systemic rheumatic diseases. *Acta Reumatol Port* 42(3)**,** 232-239.

SER (2014). "Manual SER de enfermedades reumáticas. Sexta edición", (ed.) E. Elsevier.).

Smolen, J.S., Landewé, R.B.M., Bijlsma, J.W.J., Burmester, G.R., Dougados, M., Kerschbaumer, A., et al. (2020). EULAR recommendations for the management of rheumatoid arthritis with synthetic and biological disease-modifying antirheumatic drugs: 2019 update. *Annals of the Rheumatic Diseases* 79(6)**,** 685. doi: 10.1136/annrheumdis-2019-216655.

SNS, S.N.d.S.I., Autoridade Nacional do Medicamento e Produtos de Saúde, I.P. (2021). *Medicamentos biossimilares* [Online]. Available: https://www.infarmed.pt/web/infarmed/entidades/medicamentos-uso-humano/monitorizacao-mercado/benchmarking/benchmarking-hospitalar/medicamentos-biosimilares [Accessed].

Sriranganathan, D., Segal, J.P., and Garg, M. (2021). Biologics recommendations in the ECCO guidelines on therapeutics in Crohn's disease: medical treatment. *Frontline Gastroenterology***,** flgastro-2021-101881. doi: 10.1136/flgastro-2021-101881.

Torres, J., Bonovas, S., Doherty, G., Kucharzik, T., Gisbert, J.P., Raine, T., et al. (2020a). ECCO Guidelines on Therapeutics in Crohn's Disease: Medical Treatment. *Journal of Crohn's and Colitis* 14(1)**,** 4-22. doi: 10.1093/ecco-jcc/jjz180.

Torres, T., Ferreira, A., Ferreira, P., Henriques, M., Leite, L., Magina, S., et al. (2016). Portuguese Position Paper on the Use of Biosimilars in Psoriasis. *Acta Med Port* 29(9)**,** 574-577. doi: 10.20344/amp.8118.

Torres, T., Tavares Bello, R., Paiva Lopes, M.J., Menezes Brandão, F., Ferreira, A., Ferreira, P., et al. (2020b). Portuguese recommendations for the treatment of psoriasis with biologic therapy. *Eur J Dermatol* 30(6)**,** 645-654. doi: 10.1684/ejd.2020.3945.
